# The Nutrition Literacy Assessment Instrument for Italian Subjects, NLit-IT: Exploring Validity and Reliability

**DOI:** 10.3390/ijerph18073562

**Published:** 2021-03-30

**Authors:** Virginia Vettori, Chiara Lorini, Heather D. Gibbs, Francesco Sofi, Vieri Lastrucci, Gino Sartor, Ilaria Fulvi, Duccio Giorgetti, Giuseppe Cavallo, Guglielmo Bonaccorsi

**Affiliations:** 1 Department of Health Sciences, University of Florence, 48 Morgagni Blvd, 50134 Florence, Italy; chiara.lorini@unifi.it (C.L.); vieri.lastrucci@unifi.it (V.L.); ilaria.fulvi@stud.unifi.it (I.F.); giuseppe.cavallo@unifi.it (G.C.); guglielmo.bonaccorsi@unifi.it (G.B.); 2 Department of Dietetics and Nutrition, University of Kansas Medical Center, 3901 Rainbow Blvd/MS4013, Kansas City, KS 66160, USA; hgibbs@kumc.edu; 3 Department of Experimental and Clinical Medicine, University of Florence, 50134 Florence, Italy; francesco.sofi@unifi.it; 4 Unit of Clinical Nutrition, University Hospital of Careggi, 50134 Florence, Italy; 5 Don Carlo Gnocchi Foundation Italy, Onlus IRCCS, 50134 Florence, Italy; 6 Global Health Center, Meyer University Hospital, 24 Gaetano Pieraccini Blvd, 50134 Florence, Italy; 7 School of Specialization in Hygiene and Preventive Medicine, University of Florence, 48 Morgagni Blvd, 50134 Florence, Italy; gino.sartor@unifi.it (G.S.); duccio.giorgetti@unifi.it (D.G.)

**Keywords:** cross cultural adaptation, food appearance, dietetics, obesity, malnutrition, food security, misinformation, food literacy, nutritional literacy

## Abstract

The aim of this study was to test validity and reliability of the adapted version of the Nutrition Literacy Assessment Instrument (NLit) for Italian people (NLit-IT). An observational cross-sectional study was conducted, involving a convenience sample of adults (*n* = 74). To explore the validity of the tool, we considered both diet quality as an outcome of NL, and health literacy (HL) as a construct that presents similarities and differences with NL. Diet quality was measured by adherence to the Mediterranean Diet (Med diet) through the validated Mediterranean Diet Literature-based adherence score (MEDI-Lite). The relationship between NL level and adherence to Med diet was assessed by linear regression analysis and computing correlations between NLit-IT and MEDI-Lite scores (Spearman’s Rho). Additionally, we evaluated the correlation between NLit-IT score and the level of HL (Spearman’s Rho). Internal consistency and reliability were measured by Cronbach’s alpha and intraclass correlation coefficient (ICC) respectively. Internal consistency (ρT = 0.78; 95% CI, 0.69–0.84) and reliability (ICC = 0.68, 95% CI, 0.46–0.85) were confirmed. In addition, NLit-IT total score was correlated with MEDI-Lite score (Rho = 0.25; *p*-value = 0.031) and multivariate regression analysis confirmed that NL significantly contributed to MEDI-Lite score (R^2^ = 0.13; β = 0.13; *p*-value = 0.008). There was no significant association between the level of HL and NL. In conclusion, NLit-IT showed validity and reliability as a measure of NL for Italian people.

## 1. Introduction

The concepts of nutrition (or nutritional) (NL) literacy, food literacy (FL), and the extensively described concept of health literacy (HL) probably share the same origin and are based on the same idea of increasing the degree to which individuals and groups can access and use specific information needed to make health decisions that benefit the community [1]. In particular, the term of HL was first introduced in the United States in the 1970s by Scott K. Simonds, and referred to the capacity of people to read and comprehend written health materials [2]. Over time, the HL concept expanded and referred to several conceptualizations which overall describe several factors that affect individuals’ ability to access, understand, and use health information coming from multiple sources. In 2012, Sørensen et al. [3] developed an integrated model of HL, which summarizes its evidence-based dimensions and responds to an exhaustive definition of the concept. According to Sørensen, HL is related to individuals’ knowledge, motivations, and skills necessary to access, understand, evaluate health information, and put health information into practice in order to express judgments and make decisions in everyday life regarding health, prevention, and health promotion in order to maintain and improve the quality of life for the whole course of life.

The terms of FL and NL emerged later in the literature and, in general, they refer to a set of knowledge, competencies, and abilities that are necessary for people to use information regarding food and nutrition in order to achieve and preserve a healthful diet [4,5,6,7,8]. In particular Rosas et al. [9,10] highlighted the meaning of the concept of FL and they also provided a framework named “Food Literacy Wheel” that describes the construct definition, in addition to determinants, and influential factors of FL [10].

The relationship between the constructs of HL, FL, and NL is complex and, in the literature, there are some attempts to clarify these connections [6,8,11,12,13]. Some authors agreed that the characterization of NL and FL as subcategories of HL is inaccurate [6,8,12], and probably traditional conceptualizations of HL have often overlooked the complexities of the literacy skills related to the field of food and nutrition [6,8]. Additionally, definitions of HL have not discussed the impact that individuals’ food choices, eating behaviors, and decisions have on the achievement of personal and others’ nutritional health status and a more sustainable food system [5,7,8,14,15].

With regard to the comparison between NL and FL, the core elements of the two constructs partially differ since the NL definitions mostly involve nutritional information and individuals’ capacity or interest in relation to accessing and using such information in order to maintain nutritional health status [4]. These topics are also included in the concept of FL, but most FL definitions consider as core construct elements the relationship between people and food (or food system) and the capacity to use food responsibly [8,15]. Additionally, conceptual boundaries of FL are wider [8]. As argued by Vettori et al. [8], NL could be considered as a specific part of a multifaceted concept that can be called “food and nutrition literacy” (FNL) that is realized when people can eat food which satisfies personal and others’ nutritional health requirements and contributes to a sustainable food system. Despite this characterization, several authors refer only to some features of the broader concept of FNL and describe in detail specific aspects of the NL construct; for example, the interaction between patients and professionals in dietetic and nutritional fields which have a key role in influencing individual’s NL, since they disseminate information during doctor appointments [14,16]. Several consequences of NL have been identified in literature and they generally constitute a group of health-related outcomes which can influence population health status and dietary habits [8,17,18,19,20,21,22,23,24]. In the literature, there are some attempts to adequately evaluate the NL dimensions but few data concerning literacy in the fields of food and nutrition are available worldwide [13,20,23,24,25,26,27,28,29,30]. In the Italian national context, the discussion concerning the concept of NL is almost completely absent; however, there are some examples of tools which specifically refer to the concept of FL [29] or they have been developed and validated for specific population targets [30]. Analogously, in the international context there are some examples of tools developed for specific population targets (such as, young adults, elderly individuals, children) or that have never been culturally adapted to contexts different from the original [14,20,23,27]. In the literature there are some exceptions. Recently, Monteiro et al. [31] assessed the level of NL in a large sample of Portuguese people, although they did not evaluate the reliability of the instrument used.

Gibbs et al. [26] developed and validated a novel assessment tool responding to the need for a valid measure to assess the level of NL in the general population and they also explored its validity considering a cultural context different from the original [13].

The Nutrition Literacy Assessment Instrument (NLit) was developed, validated, and refined by Gibbs et al. [13,24,25,26,32,33,34,35,36] in order to proficiently measure all the aspects embraced by the NL construct. In particular, NLit covers the following subscales: “Nutrition and Health”, that measures reading comprehension of the summarized Dietary Guidelines for Americans and includes 10 items; “Energy Sources in Food”, that measures knowledge of the macronutrient sources in food (10 items); “Household Food Measurement”, that measures identification of recommended portions (9 items); “Food Label and Numeracy”, that measures the ability to apply information obtained from the nutrition facts panel (10 items); “Food Groups”, that measures ability to classify foods by nutrition category and includes a list of 16 items; and “Consumers Skills”, that measures the ability to navigate food products to make healthy food choices (9 items).

NLit originated in the U.S. context with the aim to develop a tool for assessing patients’ knowledge and competencies in the field of nutrition education [32,33,34] and it was subsequently adapted to different contexts [13,24,25]. The original version identifies three levels of NL: likelihood of poor NL (score ≤ 44), possibility of poor NL (score 45–57), and possibility of good NL (score ≥ 58). One point was given for each correct answer.

From a public health nutrition perspective, NL could help to identify individuals with a risk of low diet quality. Unhealthy eating habits are among the main reasons of the low diet quality that largely characterizes the American population [37,38,39]. These health problems have been brought into focus even more with the emergence of COVID-19, as people living with diet-related chronic conditions and diseases are at an increased risk of severe illness from the novel coronavirus [38].

Similar problems heavily affect the Italian population as well, and some current eating behaviors decrease adherence to the traditional Mediterranean diet (Med diet) compared with the past [40]. Med diet is considered a healthy diet as it is rich in vegetable and fruits, whole grains, and lean proteins (like lean meat, fish, and dried beans) and is associated with several positive health outcomes [41,42,43]. Progressive departure from the Med diet is a harbinger of negative health consequences.

The purpose of our research was to provide a valid NL assessment tool for Italians, and we focused on the validation of the Nutrition Literacy Assessment Instrument (NLit) as adapted for the Italian population, considering both the cultural context of food and nutritional information as well as language. We explored the validity of the tool using two approaches. First, because a better quality diet is the expected outcome of NL, we evaluated the relationship between NL and diet quality as measured by adherence to the Mediterranean Diet. The second approach was based on studying the relationship between the constructs of NL and HL, that some authors consider to be overlapped [11]. Therefore, specific aims of this study were: (i) to adapt the original version of the NLit to the Italian population, and to estimate the validity and reliability of the tool by testing the Nutrition Literacy Assessment Instrument for Italian subjects (NLit-IT) in a convenience sample of Italian adults, considering both the level of adherence to the Mediterranean Diet (ii); and the level of HL. This last objective allowed for a comparison of concepts and to establish whether the concepts of NL and HL are two distinct constructs or not (iii).

## 2. Materials and Methods

This is an observational, cross-sectional study. All the data were collected between and June and July 2020. The University Research Ethics Commission approved the study (Prot. n. 0082818—UOR: ARRI—Classif. IV/5) and all the adopted procedures were in accordance with the ethical standards described in the Declaration of Helsinki. The study was divided in two phases: (1) cultural and linguistic adaptation, and (2) instrument validity assessment (cross-sectional phase).

### 2.1. Cultural and Linguistic Adaptation, Phase 1

The translation of the instrument into Italian language was performed by independent pairs of translators (two native Italian speakers and two native English speakers) who anonymously translated and later proofread the results of the back-translation process. Finally, a committee of three members of the research group reviewed and revised the different translations and decided on the most appropriate one.

The subsequent step was the cultural adaptation. The aim of this phase was to ensure that foods and meals presented in the adapted version of the instrument were familiar to the target population and appeared as typical dishes of the Italian daily diet. This procedure was in accordance with the previous example of cultural adaptation of the instrument [13].

A focus group involving five experts (two accredited nutritionists and three experts in the field of public health and public health nutrition) was formed to select items needing adaptation and determine criteria to perform substitutions for American food items in the original tool. The two nutritionists proposed the modified version of items selected and the three experts scored the proposals. A five-point Likert scale was considered for each criterion and the version which gained the highest score was finally chosen. The criteria selected for item adaptation were: (a) equivalence in terms of nutritional composition (that is the correspondence in terms of macronutrients, and energy intake from the original version); (b) selection of dishes commonly consumed by the Italian population; and (c) visual appearance of food. 

Calculations of nutritional values were based on the U.S. Department of Agriculture (USDA) database [44] for the original version; and the Italian National Food Research Institute (ex-INRAN, renamed CREA) [45] for the adapted items. Nutritionists also referred to the latest recommended nutrient intake levels for the Italian population [46], and they replaced Imperial units of measurement (ounces, oz) with European grams (g) (1 oz corresponds to 28.35 g).

An example of adaptation is provided as follows: “8 oz reduced-fat milk, 2 ounces sausage patty” was replaced with “a rosetta bread and mortadella, 40 g” that has similar energetical and nutritional values of the original option.

In regard to the subscale “Household Food Measurement”, in addition to adapting original items with more familiar recipes, all the dishes were prepared, and the correspondent pictures added (Raw format saved). In regard to the fourth subscale “Food Label and Numeracy”, the researchers agreed to replace the original food label since in the Italian context “maccheroni” is generally a homemade dish.

Lastly, the research group decided to change several pictures of the subscale “Consumers Skills” because they appear very different from the national packages used in the national food trade system. Pictures from public subscale photos database (CCO free photos) were searched and modified in order to maintain both the correspondence with the original version tool and the similarity with national food packages.

In line with what has been proposed by Gibbs et al. [13], cognitive interviews were conducted with the aim of evaluating both clarity of language and familiarity with food items. Interviews were performed by a member of the research team with five Italian adults. Interviews foresaw an open dialogue, collecting and discussing the participants’ opinions and interpretations as they answered the questions [13]. These opinions helped to refine the Italian adapted version of NLit.

An open discussion with the original NLit creator (Professor Dr Heather D. Gibbs) was carried out about the entire methodological approach in order to ensure that changes did not alter the intended NLit construct.

### 2.2. Instrument Validity and Reliability, Phase 2

For validity testing, a convenience sample of *n* = 74 Italian adults was recruited based on specific selection criteria, and the online questionnaire containing the NLit-IT was administered. A convenience recruitment was carried out: the project was promoted within the university campus through flyers and posters. Two members of the research team verified the inclusion and exclusion criteria for each subject interested in participating in the study. Inclusion criteria were the following: 18–69 years old and Italian speaking. Exclusion criteria included cognitive impairment, severe psychiatric diseases, and end-stage diseases. Subjects receiving nutritional treatment for gastrointestinal and/or renal disorders were also excluded because these conditions require specific diet recommendations that may differ from healthy diet guidelines for the general population.

The two researchers informed each participant about the study and its objectives via verbal contact. Each subject who verbally confirmed to participate received by email an information sheet signed by the person in charge of the study, an invitation to participate, and an informative letter. Subjects also received the links to access the questionnaires and a letter containing a code that they needed to enter before starting the compilation of the questionnaires. Only the participants knew the code, so that data were collected in a completely anonymous format. Questionnaires included the adapted version of the NLit-IT; the MEDI-Lite, to assess the adherence to the Med diet [47]; and HLS-EU-Q6 Italian version, to assess the general Health Literacy [48,49,50,51,52].

The sample size was decided on the basis of a previous example of cultural adaptation of NLit [13], that was for Spanish-Speaking Latinos context in which the content validity and reliability were confirmed [13,26]. The NLit-IT instrument was also assessed by counting the number of missing answers per item.

### 2.3. Measures

As Gibbs et al. [26] observed, there are no international standards for measuring NL, and diet quality was expected to be related to scores of the NL measure. In fact, as previously described in the text, diet quality represents a potential outcome of NL [8,17,18,19,20,21,22,23].

In the Italian national context, the quality of diet should be provided by adherence to the Med diet, which is the reference dietary pattern of the national dietary guidelines for Italians and is effective in maintaining health [41,42,43]. In this study, adherence to the Med diet was assessed using the validated Mediterranean Diet Literature-based adherence score (MEDI-Lite) [47].

The 6-item European Health Literacy Survey Questionnaire (HLS-EU-Q6) is a tool projected and used by the European Health Literacy Consortium to measure and compare the general HL in eight different countries [53]. In our research, the Italian version of the instrument [50,51] was administered to assess similarities and differences between the construct of NL and HL. We also assessed weekly consumption of soft drink and fast-food consumption since literature anticipated an association between these variables and NL construct [8]. MEDI-LITE and HLS-EU-Q6 are both valid tools to assess the level of adherence to the Med diet and the level of HL, respectively; additionally, they have the advantage of being quickly administered.

All the tools were administered and completed online by receiving links to access questionnaires that included items of demographic characteristics, NLit-IT, adherence to Med diet (MEDI-Lite), level of HL (HLS-EU-Q6), soft drink and fast-food consumption. Participants were sent another link from the researchers one month later to complete NLit-IT a second time for Test-retest purposes.

#### 2.3.1. Mediterranean Diet Literature-Based Adherence Score (MEDI-Lite)

The questionnaire [47] assesses the level of adherence to the Med diet by means of a score that varies from 0 (low adherence) to 18 (high adherence). It considers the daily or weekly consumption of the following food items: fruit, vegetables, cereals, legumes, fish and fish-products, meat and meat-products, dairy products, and the consumption of alcohol and olive oil. To determine the final score for each food group of the Med diet (fruit, vegetables, cereals, legumes, fish and fish-products), a score of 2 is assigned for the highest category of consumption, 1 for the intermediate, and 0 for the lowest. Conversely, for food groups that are not characteristic of the Med diet (meat and dairy products) a score of 2 is assigned for the lowest category of consumption, 1 for the intermediate, and 0 for the highest. Regarding alcohol, the score of 2 is given in case of 1–2 alcoholic units/per day of consumption, 1 and 0 is assigned for the lowest and highest category of consumption respectively. Finally, regarding olive oil, a score of 2 is attributed for regular consumption, 1 for frequent, and 0 for occasional intake. The final score is obtained by the sum of all values.

#### 2.3.2. 6-Item European Health Literacy Survey Questionnaire (HLS-EU-Q6)

The HLS-EU-Q6 represents the short-short form of the 47-item questionnaire for the assessment of general HL, which was used in the European survey led in eight countries in 2015 [53,54]. It was developed considering the Sorensen’s conceptual model of HL [3] and was recently validated in Italian context [50,51]. Scores for the instrument were calculated on the basis of a four-point Likert scale for each of the six items (from 1 for “very difficult”, to 4 for “very easy”). The final score ranges from 1 to 4 and represents the mean calculated if at least five of the six items have been completed. According to the final score, three possible levels of HL have been defined: inadequate HL (1–2); problematic HL (2–3); sufficient HL (3–4).

#### 2.3.3. Sociodemographic Characteristics, Body Mass Index (kg/m^2^) (BMI), Soft Drink, and Fast-Food Consumption

The following sociodemographic characteristics were collected: gender, age, educational level, and financial situation; and regarding anthropometric measurements, self-reported height and weight. Data related to educational level and financial situation were collected by following the classification in use of the National Surveillance Progress by Local Health Units towards a Healthier Italy (PASSI) [55], which has been active in Italy since 2006. The body mass index (BMI) was calculated using the weight in kilograms divided by the square of the height in meters (kg/m^2^); beginning from a value ≥ 25 kg/m^2^, BMI identifies overweight and obese subjects [56]. Potential eating behaviors that are influenced by NL including soft drink and fast-food consumption [8] were also investigated using dichotomous items.

### 2.4. Data Analysis

Data analysis was performed using RStudio software 1.3.1093 (University of Padua, Padua, Italy). For all of the analyses, a *p*-value equal to or less than 0.05 was considered significant. Data was presented as percentage or mean and standard deviation (SD). Internal consistency was assessed by means of Cronbach’s alpha of the questionnaire for both the total score and each subscale. To assess test-retest reliability, intraclass correlation coefficients (ICCs) were calculated for the total score and for each subscale.

Associations between NLit-IT scores and MEDI-Lite score, HLS-EU-Q6 score, BMI (kg/m^2^), sociodemographic characteristics, soft drink, and fast-food consumption were assessed by computing correlations (Spearman’s Rho) or Kruskal-Wallis test.

Multivariate linear regressions analysis was performed including as dependent variable MEDI-Lite score and as independent variables those significantly associated with the outcome variable in the univariate analysis. The final model was obtained using the backward stepwise procedure.

## 3. Results

A slightly higher portion of the total sample (*n* = 74) was female (56.8%); the mean age was 38.8 years old (95% CI, 35.8–41.8; SD, 13.1); and the majority had an education level corresponding to secondary education diploma or higher (75.6%). Characteristics of the sample were detailed in Table 1.

### 3.1. Phase 1

Results from cognitive interviews indicated all NLit-IT items were relevant and clear. In fact, the maximum of missing answers per item confirmed the easiness of comprehension and it was 4.7%; thirteen items had 1.6% missing, item 54 had 3.1% missing, and item 44 had 4.7% missing.

The researchers agreed to maintain all the items of the original NLit and to perform only adaptations of some of them; in so doing, NLit-IT has the same structure of the original construct.

Regarding the first part of the subscale “Nutrition and Health”, the experts agreed that it did not need adaptation since there is good agreement between basic dietary guidelines for Americans and those for the Italian population. Instead, two items of the second part of this subscale needed adaptation.

Two subscales (the third and the fourth subscale) were entirely adapted on the basis of the accordance among members who carried out the cross-cultural adaptation (Please see the paragraph Cultural and Linguistic Adaptation). Specifically, all the items of the subscale “Household Food Measurement” were changed to improve the cultural relevance of the subscale. The subsequent subscale “Food Label and Numeracy” was completely adapted, too. By proceeding in the adaptation of this subscale, the label of the original instrument was replaced with the label of a typical Italian Sunday lunch that is “lasagne alla bolognese”, commonly bought as ready-to-eat, and the calculations for the modification of the proportions were executed.

We also changed an item of the subscale “Food Groups”, and we replaced all the photos of the subscale “Consumer Skills” except for the items 3 and 6, that maintained the images of the original tool. Lastly, six more items were replaced and all changes to food items were shown in Table 2.

### 3.2. Phase 2

The mean scores for MEDI-Lite, HLS-EU-Q6, and NLit-IT were 10 (95% CI, 9.42–10.58; SD, 2.55); 2.6 (95% CI, 2.5–2.7; SD, 0.48); and 49 (95% CI, 47.73–50.27; SD, 5.59), respectively.

The reliability of NLit-IT score was good (ICC = 0.68; 95% CI, 0.46–0.85) and the total score of the questionnaire had the Cronbach’s alpha of 0.78 (95% CI, 0.69–0.84). ICC and Cronbach’s alpha of the six subscales were shown in Table 3.

The associations found between NLit-IT scores and variables considered were shown in Table 4.

The total score of NLit-IT had positive significant associations with MEDI-Lite score (Rho = 0.25; *p*-value = 0.031) and education (median, 50.5; SD, 5.55; *p*-value = 0.005).

Subscale 1 “Nutrition and Health” of NLit-IT had significant associations with BMI (kg/m^2^) (Rho = −0.26; *p*-value = 0.024) and education (median (Less than High school or General Equivalency Diploma), 9; median (General Equivalency Diploma), 6; median (High school), 6; median (Bachelor’s degree or University Diploma or higher) 9; *p*-value = 0.009). Subscale 4 “Food Label and Numeracy” was significantly associated with education too (median (Less than High school or General Equivalency Diploma), 8; median (General Equivalency Diploma), 6; median (High school), 6; median (Bachelor’s degree or University Diploma or higher) 10; *p*-value = 0.009). Subscale 3 “Household Food Measurement” had positive significant association with MEDI-Lite score (Rho = 0.27; *p*-value = 0.019). Soft drink consumption was associated with subscale 2 “Energy Sources in Food” (median (Yes), 8; median (No), 9; *p*-value = 0.02) and 5 “Food Groups” (median (Yes), 12; median (No), 13; *p*-value = 0.01) too. Additionally, subscale 5 “Food Groups” was associated with fast-food consumption (median (Yes), 12; median (No), 13; *p*-value = 0.03). There were no significant associations between NLit-IT scores and HLS-EU-Q6 score. Univariate linear regressions performed showed that the variables significantly associated with MEDI-Lite score were NLit-IT score, age, soft drink, and fast-food consumption and they were so included for multivariate linear regressions. In the final model, NLit-IT score and age confirmed positive significant associations with MEDI-Lite score (R^2^ (NLit-IT score) = 0.13; R^2^ (age) = 0.05; β = 0.13; *p*-value = 0.008). Multivariate regressions were shown in Table 5.

## 4. Discussion

In the present research, reliability and validity of the translated and adapted questionnaire for the assessment of NL in Italian context (NLit-IT) were assessed. Results obtained proved internal consistency (ρT = 0.78; 95% CI, 0.69–0.84) and reliability (ICC = 0.68, 95% CI, 0.46–0.85) of NLit-IT. NLit-IT score was positively associated with diet quality (MEDI-Lite score) (*p* < 0.05) and education (*p* < 0.01). Additionally, multivariate linear regression analysis confirmed that NLit-IT score, along with age, contributed to predict MEDI-Lite score (*p* < 0.01).

In line with previous validation studies [13,26], diet quality was considered as a convergent construct of NL and the scores of MEDI-Lite and NLit-IT were expected to trend in the same direction. Evidence from literature suggests that diet quality and healthy eating behaviors are influenced by NL [8]. Individuals’ NL originates from information exchange with experts, peers, parents or caregivers, and it is also influenced by the context. This process may give rise to skilled individuals [17,58,59] and lead them to make healthier food choices as well as developing and maintaining healthy dietary habits [17,18,19,22,23]. Most recently, Ahmadpour et al. [60] investigated this relationship and focused their attention in youth. These authors attributed a central role of NL as a key instrument for health policies oriented to promote healthful diets in elementary school children.

In the Italian context, one of the best indicators of diet quality is represented by adherence to the Mediterranean Diet, since it proved effective in reducing the risk of overall as well as cardiovascular disease mortality [61,62]. Other evidence from the literature confirmed the role of this dietary pattern in preventing overall cancer mortality and neurodegenerative diseases [41,42]. Most recently, research confirmed the role of this dietary pattern in the prevention of cardiovascular diseases (such as blood pressure, dyslipidemia, and diabetes) and cancer as well as brain function (e.g., Parkinson’s disease) [63] and mental health conditions (e.g., major depression, and anxiety) [64]. Additionally, a very recent international research study suggested that increased adherence to the Mediterranean Diet seems to be positively related with increased physical activity in youth [65].

Along with the Mediterranean Diet, we found an association between NLit-IT score and education: this is in line with the literature that discussed the relationship between education and health [3] or nutritional literacy [26,35] and, in the case of NL, it embraces a set of individuals’ knowledge and competencies that develop over the course of life and could be regarded as an outcome of nutritional health education.

The other covariate together with NLit-IT score that significantly contributed to MEDI-Lite score was age; however, the strength of this association is very small and in line with what has been already discussed in literature where the adherence to this diet is more closely associated with older age [66].

Mediterranean diet has been recognized by UNESCO as a cultural heritage of humanity and all the Mediterranean populations could easily benefit from the specific characteristics of their traditional diet. Despite this, some worrying data showed that Italian people are moving away from this diet [67], including the youngest [68,69].

In particular, from literature emerges a lower-than-recommended intake of all food categories included in the Mediterranean diet pyramid, along with excess in sweets, red and processed meats, and a widespread sedentary lifestyle [67]. In line with this evidence, our sample, that showed an intermediate level of NL (49 score), had a level of adherence to the Mediterranean diet far from high (10 score).

This mediocre level of adherence to the Mediterranean diet is in line with results obtained through a large online survey conducted in a sample of 1820 individuals who completed the web version of the MEDI-Lite score during the year 2019 [42]. These results also suggested that female gender, advanced age (>45 years), and higher education level were associated with a higher probability of having the highest level of adherence and that some sub-groups (males, younger individuals, and those with lower levels of education) need to improve their eating behaviors for reaching higher adherence scores to the Mediterranean Diet.

Regarding the comparison between HL and NL, our results suggested that there is not a significant association between HLS-EU-Q6 score and NLit-IT score in line with what has already been published [13]. Indeed, it was observed that NL requires knowledge, competencies, and skills that are not measurable in the same way used by HL tools [13].

The results obtained through the present research corroborate the point of view proposed in the literature that HL is separate from NL by conceptual boundaries. Like other validation studies [13], NLit-IT was not only linguistically, but also culturally adapted. A group of experts in the field of nutrition, dietetics, and public health drove the adaptation phase, maintaining the same construct of the original instrument (same subscales and same number of items), and at the same time including food dishes which are familiar for Italian people. This seems to produce an adequate level of comprehension, as evidenced by the low level of missing answers per item (lower than 5%).

Our results proved that the internal consistency and the reliability of the entire NLit-IT were good and higher than the single subscale, this testifies that it is advisable the administration of the entire tool instead of single subscale to assess the entire construct of NL.

Some investigations conducted in the national context anticipated a medium level of procedural knowledge in the field of nutrition and health [40]; the same authors also discussed that this kind of knowledge was significantly associated with adherence to the Mediterranean dietary pattern and a lower prevalence of obesity [40].

Other national research [70] has focused on children, which represent an important segment of the population at risk of low diet quality since unhealthy diet behaviors and a sedentary lifestyle are common in this age group [71]. These authors [70] described an innovative educational program based on the approach “learning through playing”, which seems to be effective in children considering nutritional knowledge increased after the intervention.

Despite this evidence, data provided by the literature came from questionnaires that referred to a construct different from NLit. In one of the previously cited studies [40], the questionnaire included some subscales that did not referto the field of nutrition or dietetics and it missed some subscales included in the NLit such as “Household Food Measurement” and “Food Label and Numeracy”.

The study presented in this manuscript has some limitations. First, it was subjected to common survey limitations such as self-reported data and social desirability, so that future studies in the national context should consider larger and representative, instead of convenience, samples. Some authors highlighted limitations in this approach such as inherent bias related to self-report and measurement errors related to methodology [72]. Additionally, although NLit-IT has shown good internal consistency and reliability, values are lower for the subscale 3 “Household Food Measurement” and future studies aiming to deepen validity and reliability of NLit-IT should consider other factors that influence reliability. Finally, our results suggest it is reasonable to think higher levels of education are associated with higher levels of NL and vice versa; however, data related to educational level was collected in line with the Italian National Surveillance PASSI [55] that does not allow to distinguish between the highest educational degrees which are all grouped under the same heading “Bachelor’s degree or University Diploma or higher”. These differences about the relationship between the highest levels of education and NL still need to be explored.

## 5. Conclusions

Although a very large amount of information about food and nutrition is available to consumers who can access it, they often struggle to recognize evidence-based information and effectively manage their diet. Thus, there is a need to develop and implement interventions in order to increase the public’s NL level. The first step is represented by establishing valid instruments to measure the NL dimensions. The research presented in this manuscript is based on this perspective and represents the first attempt to study validity and reliability of adapted version of NLit for Italians.

Furthermore, the administration of validated instruments to measure NL could have a vital role in clinical fields in the development of an individual’s awareness of NL. This awareness could help professionals guide their patients in the achievement of healthful diet.

In line with the study’s purpose, specific goals have been achieved. We adapted not only linguistically, but also culturally, the NLit tool, and we estimated reliability and validity of the adapted version NLit-IT. Cultural adaptation of NLit was performed by experts in the field of nutrition and dietetics and public health nutrition. Thanks to this methodological approach, we obtained a validated tool which contains items, represented by foods and dishes, that are familiar to the general Italian population, without altering the intended construct of the original tool. Further studies with the same objective of the present research and conducted in countries different from US or Italy should consider and perform this phase of adaptation. We recommend to conduct cultural adaptation of the tool using official dietary guidelines for the target population to perform substitutions of foods and dishes. Secondly, we recommend to maintain correspondence in terms of macronutrients, and energy intake from the original version.

Regarding the relationship between HL and NL investigated in the present study, data analysis conducted supported the idea that they could be considered as distinct concepts.

In addition, results obtained showed that internal consistency and reliability of the tool are good, and there is a significant association between NLit-IT score and MEDI-Lite score. Specifically, multivariate linear regression analysis suggested that NLit-IT score positively predicted, though slightly, the adherence to Mediterranean Diet. These results are in line with findings of the original NLit validation; and this consistency across cultural contexts increases the validity of the NLit tool and suggests it could be applicable and similarly adapted to other cultural contexts.

We argue that this research could contribute in filling the gap concerning NL levels of the Italian population by proving a valid instrument to measure this construct. Our findings also provide an example of adaptation of the NLit in a population that present differences and distinctiveness regarding cultural and environmental background with respect to the US context.

## Figures and Tables

**Table 1:** Characteristics of the sample that participated in validity testing of Nutrition Literacy Assessment Instrument for Italian Subjects (NLit-IT) (*n* = 74).

Characteristics	Mean (SD) or *n* (%)
Age	38.8 (13.1)
Gender	
Female	42 (56.8%)
Education	
Less than Secondary education Diploma or General Equivalency Diploma	7 (9.5%)
General Equivalency Diploma	11 (14.9%)
Secondary education Diploma	22 (29.7%)
Bachelor’s degree or University Diploma or higher	34 (45.9%)
Financial situation	
Quite or very difficult	15 (20.3%)
Quite or very easy	52 (70.3%)
Refusal response	7 (9.4%)
BMI (kg/m^2^)	23.5 (3.1)
Soft drink consumption	
Yes	20 (27%)
Fast-food consumption	
Yes	17 (23%)
NLit-IT score	49 (5.6)
NLit-IT score Retest *	51.9 (6.9)
MEDI-Lite score	10 (2.6)
HLS-EU-Q6 score	2.6 (0.5)

* *n* = 61 participants completed the second administration of NLit-IT (Test-retest).

**Table 2:** Cultural adaptation and substitution of the original instrument items for Italian context.

Original Food Items	Final Version Food Items	Comparison and Substitution on the Basis of: Macronutrient Contents of Proteins (P), g; Fats (F), g; Carbohydrates (C), g; and Energetics Value (E, kcal)				
3 oz hamburger on wheat bun, 20 potato chips, 8 oz lowfat milk 1 cup of spaghetti with sauce, 1 slice garlic bread, 8 oz lowfat milk 3 oz skinless chicken, 1 cup steamed green beans, 8 oz lowfat milk 4 oz pork chop, ½ cup steamed white rice, 8 oz lowfat milk From the domain “Nutrition and Health”, answers A, B, C, D, item 6	100 g di filetto di bovino, 80 g di patate arrosto, mezza fetta di pane, 125 g di yogurt magro 1 piatto di spaghetti con il ragù di carne, 1 cornetto alla crema, 125 mL di yogurt magro 90 g di petto di pollo, 150 g di fagiolini cotti, mezza fetta di pane, 125 mL di yogurt magro 100 g di carne di maiale (lombo), 60 g di riso, 125 mL di yogurt magro	Original food items				
			P (g)	F (g)	C (g)	E (kcal)
		a.	27	17.2	62.8	497.7
		b.	12	9	37	279
		c.	23.3	0.7	4.8	136
		d.	27.3	22	38.5	457
		Adapted version items				
			P (g)	F (g)	C (g)	E (kcal)
		a.	29.1	9.8	42.8	362.7
		b.	10.9	4.7	55.9	289.8
		c.	23.2	0.8	16.7	170.3
		d.	28.9	11.2	53.7	416
canned tomato soup frozen corn From the domain “Nutritional and Health”, answers A, B, item 10	sugo pronto di pomodoro piselli surgelati	Original food items				
			P (g) ^	F (g) ^	C (g) ^	E (kcal) ^
		a.	1.5	0.4	15.2	66
		b.	2.5	0.7	19.2	81
		Adapted version items				
		a.	1.4	0.2	4.5	27
		b.	5.5	0.7	6.5	66
peanut butter From the domain “Energy Sources in Food”, answer D, item 4; answer B, item 5	crema spalmabile di nocciole e cacao	Original food items				
			P (g) ^	F (g) ^	C (g) ^	E (kcal) ^
			25	50	21,9	594
		Adapted version items				
			6.9	31	55	534
c 8 oz reduced-fat milk, 2 ounces sausage patty d 8 oz reduced-fat milk, 2 slices bacon From the domain “Energy Sources in Food”, answers C, D, item 7	c una rosetta da 50 g, con 40 g di mortadella d 30 grammi di pane integrale, con 2 fette di prosciutto crudo e del formaggio spalmabile	Original food items				
			P (g)	F (g)	C (g)	E (kcal)
		c.	27.2	20	13.1	297.4
		d.	14.3	12.4	12.8	261.1
		Adapted version items				
			P (g)	F (g)	C (g)	E (kcal)
		c.	10.4	12.1	29.4	261.3
		d.	14.7	11.7	14.6	219.4
regular salad dressing From the domain “Energy Sources in Food”, answer D, item 4; answer B, item 9	maionese	Original food items				
			P (g) ^	F (g) ^	C (g) ^	E (kcal) ^
			1.3	44.5	5.9	430
		Adapted version items				
			1.1	71	1.6	650
a glass that contains 8 (eight) ounces of milk From the domain “Household Food Measurement”, item 1, item 4	(1) tazza che contiene 150 mL di yogurt	Original food items				
			P (g)	F (g)	C (g)	E (kcal)
			8	8	12	149
		Adapted version items				
			5.6	6.6	6.6	112.5
½ (one half) cup of black beans, a slice of tortilla, a glass that contains 8 (eight) ounces of milk From the domain “Household Food Measurement” *, Item 6	150 g (una scatoletta) di fagioli borlotti, una fettina di pane, 1 pomodoro, un cucchiaio di olio extravergine di oliva	Original food items				
			P (g)	F (g)	C (g)	E (kcal)
		a.	6.9	0	19.6	103.5
		b.	2	1.5	20	100
		c.	4.8	4.8	6.6	89
		Total amount	13.7	6.8	37.1	279
		Adapted version items				
		a.	6.9	0.5	10.5	90
		b.	4	0	24	120
		c.	1.2	0.2	2.8	17
		d.	0	9.1	0	78
		Total amount	12.1	9.6	37.3	305
2 slices of bread 2 slices of cheese ½ (one-half) cup of uncooked carrots From the domain “Household Food Measurement” *, Item 7	1 crostone di pane integrale 1/2 confezione di mozzarella un cucchiaino di olio extravergine di oliva 1 (una) zucchina	Original food items				
			P (g)	F (g)	C (g)	E (kcal)
		a.	10	3	40	199.8
		b.	7.5	9.6	1.1	117.9
		c.	0.5	0.1	5.8	24.6
		Total amount	18	12.7	46.9	342.3
		Adapted version items				
		a.	6.4	1	33	168
		b.	11.7	12.1	0.4	158.1
		c.	1.5	0.1	1.7	16
		d.	0	4.6	0	39
		Total amount	19.6	17.7	35.1	381.1
1 cup of rice 3 (three) ounces of salmon From the domain “Household Food Measurement” *, Item 8	2 fette di pane 125 g (1 filetto piccolo) di merluzzo	Original food items				
			P (g)	F (g)	C (g)	E (kcal)
		a.	5	3.5	38	200
		b.	16.8	10.8	3	180
		Total amount	21.8	14.3	41	380
		Adapted version items				
		a.	9	1.9	57.6	275
		b.	21.3	0.4	0	88.8
		c.	0	4.6	0	39
		Total amount	30.3	6.9	57.6	402.8

* Portion size was estimated in items 6, 7, and 8 from the domain “Household Food Measurement”. ^ Per portion 100 g.

**Table 3:** Internal consistency and reliability of NLit-IT. Internal consistency was measured using Cronbach’s alpha and reliability was tested by way of intraclass correlation coefficient (ICC) (Test-retest).

NLit-IT and Subsections	Cronbach’s Alpha (ρT) (95% CI)	Intraclass Correlation Coefficient (ICC) (95% CI)
All subsections combined	0.78 (0.69–0.84)	0.68 ^I^ (0.46–0.85)
Section 1	0.59 (0.45–0.72)	0.49 ^II^ (0.21–0.87)
Section 2	0.5 (0.28–0.64)	0.51 ^II^ (0.22–0.87)
Section 3	0.27 (0.1–0.55)	0.19 ^III^ (0.01–0.63)
Section 4	0.8 (0.76–0.87)	0.45 ^II^ (0.19–0.77)
Section 5	0.45 (0.16–0.57)	0.48 ^II^ (0.21–0.84)
Section 6	0.52 (0.29–0.66)	0.47 ^II^ (0.2–0.83)

^I, II, III^ Reliability was classified as follows: excellent (0.75–1), good reliability ^I^ (0.60–0.74), fair reliability ^II^ (0.40–0.59), and poor reliability ^III^ (≤0.40) [57].

**Table 4:** Associations between NLit-IT score (for each section and all subsections combined) and the following variables: MEDI-Lite score, HLS-EU-Q6 score, age, BMI (kg/m^2^), gender, educational level, financial situation, consumption of soft drink or fast-food.

	MEDI-Lite Score *	HLS-EU-Q6 Score *	Age *	BMI *	Gender °	Education °	Financial Situation °	Consume Soft Drink °	Consume Fast-Food °
All sub-sections combined	*p* < 0.05	*p* ≥ 0.05	*p* ≥ 0.05	*p* ≥ 0.05	*p* ≥ 0.05	*p* < 0.01	*p* ≥ 0.05	*p* ≥ 0.05	*p* ≥ 0.05
Section 1	*p* ≥ 0.05	*p* ≥ 0.05	*p* ≥ 0.05	*p* < 0.05	*p* ≥ 0.05	*p* < 0.05	*p* ≥ 0.05	*p* ≥ 0.05	*p* ≥ 0.05
Section 2	*p* ≥ 0.05	*p* ≥ 0.05	*p* ≥ 0.05	*p* ≥ 0.05	*p* ≥ 0.05	*p* ≥ 0.05	*p* ≥ 0.05	*p* < 0.05	*p* ≥ 0.05
Section 3	*p* < 0.05	*p* ≥ 0.05	*p* ≥ 0.05	*p* ≥ 0.05	*p* ≥ 0.05	*p* ≥ 0.05	*p* ≥ 0.05	*p* ≥ 0.05	*p* ≥ 0.05
Section 4	*p* ≥ 0.05	*p* ≥ 0.05	*p* ≥ 0.05	*p* ≥ 0.05	*p* ≥ 0.05	*p* < 0.01	*p* ≥ 0.05	*p* ≥ 0.05	*p* ≥ 0.05
Section 5	*p* ≥ 0.05	*p* ≥ 0.05	*p* ≥ 0.05	*p* ≥ 0.05	*p* ≥ 0.05	*p* ≥ 0.05	*p* ≥ 0.05	*p* < 0.05	*p* < 0.05
Section 6	*p* ≥ 0.05	*p* ≥ 0.05	*p* ≥ 0.05	*p* ≥ 0.05	*p* ≥ 0.05	*p* ≥ 0.05	*p* ≥ 0.05	*p* ≥ 0.05	*p* ≥ 0.05

* Spearman’s Rho was calculated; ° Kruskal-Wallis test was computed.

**Table 5:** In the first model, multivariate model of linear regression analysis was fitted setting up MEDI-Lite score as the dependent variable (*y*) and NLit-IT score, age, “soft drink consumption”, and “fast-food consumption” as independent variables (*x*) because they showed significant associations with MEDI-Lite score performing univariate linear regressions. In the final model, NLit-IT score and age were considered and they confirmed to positive significantly contribute to variability of *y*.

**First Model**			
	**Multiple R-Squared (R^2^)**	**Adjusted R-Squared**	***p*-Value**
	0.1603	0.1116	0.016
	**β**	**Standard Error**	***p*-Value**
NLit-IT score	0.1205	0.0516	0.0224
Age	0.0426	0.0223	0.0608
To consume soft drink	0.069	0.6753	0.9188
To consume fast-food	−1.099	0.6872	0.1143
**Final Model**			
	**Multiple R-squared (R^2^)**	**Adjusted R-squared**	***p*-Value**
	0.1284	0.1039	0.008
	**β**	**Standard Error**	***p*-Value**
NLit-IT score	0.1298	0.0505	0.0123
Age	0.0449	0.0218	0.0427

## Data Availability

The data are stored in a password-protected electronic archive held by the person in charge of the study. Only the person in charge of the study and the researchers of data curation, V.V., C.L., and G.B. can access the file archive.
